# Special Issue: “Structural and Thermal Properties of Polymeric Microspheres”

**DOI:** 10.3390/ma15228017

**Published:** 2022-11-14

**Authors:** Małgorzata Maciejewska

**Affiliations:** Department of Polymer Chemistry, Institute of Chemical Sciences, Faculty of Chemistry, Maria Curie-Skłodowska University in Lublin, Gliniana 33, 20-614 Lublin, Poland; mmacieje@umcs.pl

In recent years, polymeric materials have become the backbone of modern industry and technology. In the polymer family, materials with spherical shapes (porous and nonporous beads, microspheres, nanospheres, etc.) gained remarkable attention thanks to their superior inherent porosity, excellent stability, and predesignable and tunable structures. Microspheres are easy to prepare and handle; they do not possess sharp edges and may be readily used in packed beads for continuous flow operation. They are among the most effective materials due to their many separation processes. Permanent porosity, along with chemical and thermal resistance, generates significant advantages in many practical applications. They are widely used as stationary phases in different kinds of chromatography, drug delivery systems, nuclear imaging, cell culturing, specific sorbents, as supports for catalysts and sensors, as carriers in drug delivery systems, in water treatment and CO_2_ capture, etc. In comparison with alternative adsorbents, polymeric microspheres are stable throughout the whole pH range, and they can be simply functionalized and have the ability to create specific sorption spaces.

The abovementioned applications of the polymeric microspheres are strongly connected with their structural and thermal properties. Their porous structure, pore size, specific surface area, and application can be directly designed and facilely tuned by introducing specific functional building blocks. The considerable thermal resistance that is indispensable in many important techniques (e.g., gas chromatography or catalysis processes often are conducted at temperatures that significantly exceed 200 °C). Knowledge on the materials’ behavior under harsh conditions and the consequences of their decomposition is crucial to increase their service lifetime.

A detailed investigation of structural and thermal properties creates the possibility to improve the materials and achieve more effective ones. Consequently, comprehensive research focused on these features is indispensable.

This Special Issue brings together some of these mentioned aspects with a total of four original articles. A concise description of these publications, which I am honored to edit as a Guest Editor, is presented below to highlight their high academic standard.

An interesting investigation was conducted by K. Wnuczek et al. [1]. They carried out the synthesis of new polymer microspheres based on ethylene glycol dimethacrylate (EGDMA), styrene (St), and various quantities of commercial kraft lignin (L). Along with increasing the amount of lignin (from 13% w/w to 64% w/w in reference to monomers), the basic parameters of the morphology, porous structure, and thermal stability are changed. The obtained microspheres are more irregular. Their internal structure is less developed, but they possess a higher thermal resistance. This phenomenon can be connected with the presence of the aromatic rings in the structure of the incorporated lignin.

In addition to the synthesis, the carbonization of selected regular copolymeric microspheres rich in lignin (named EGDMA + St + 4L) was conducted. The carbonization process showed how the specific surface would be affected by the activator type and temperature. All of the materials obtained by carbonization possessed larger porosity parameters (S_BET_, V_TOT_) than the output polymeric sample. It turned out that sulfuric acid is the most effective surface activator for EGDMA + St + L copolymers. The carbonized materials were used as sorbents for acetylic acid sorption from its methanolic solution.

The successful incorporation of a well-liked biopolymer–lignin in a polymeric matrix is an interesting proposal in the field of more ecological and biodegradable polymeric sorbents.

A fairly complete study on the permanently porous copolymers of divinylbenzene (DVB) with diverse functional monomers was performed [2]. They were synthesized in the form of regular microspheres to obtain polymeric microspheres with a highly developed internal structure, remarkable thermal stability, and diverse functional group. N-vinylpyrrolidone, (NVP), ethylene glycol dimethacrylate (EGDMA), 4,4′bis(methacryloyloxymethylphenyl)sulphone (BMPS), 4,4′-bis(methacryloyloxymethylphenyl)methane (BMPM), and 1,4-bis(methacryloyloxymethyl)benzene (BMB) were used as functional monomers. The synthesized materials were characterized by ATR-FTIR, scanning electron microscopy, a size distribution analysis, a low-temperature nitrogen adsorption–desorption method, differential scanning calorimetry, and thermogravimetry coupled with FTIR and inverse gas chromatography. Depending on the used functional monomer, regular microspheres with a specific surface area in the range of 418–746 m^2^/g were successfully synthesized. Importantly, all copolymers with various functional groups exhibited a higher surface-area value than that of the commonly used DVB-co-ST copolymer. Moreover, the introduction of functional groups did not negatively impact the thermal behavior of the materials. All the obtained copolymers demonstrated good thermal stability. In helium, their temperatures were over 300 °C with 5% mass losses, whereas in air these values were only slightly lower. Their decomposition in both atmospheres generally took place in two partially overlapping stages and was accompanied by the evolution of the same kinds of volatile products. The basic volatile products were vinyl compounds, carbon dioxide, carbon monoxide, and water. It is important to note that the presence of the functional groups contributed to an increase in overall polarity compared to the DVB-co-ST copolymer and promoted diverse kinds of interactions. As a result, the microspheres can be possibly use in many adsorption techniques, including high-temperature processes.

An interesting contribution regarding the sorption properties of poly(divinylbenzene) microspheres modified using maleic anhydride in a Diels–Alder reaction was presented by Ronka et al. [3]. Desethyl-terbuthylazine and 2-hydroxyterbuthylazine (persistent and mobile metabolites of terbuthylazine) were used as analytes. The efficiency of their adsorption and the impact of various factors (time, temperature, and pH) on this process were investigated in a batch experiment. Four adsorption models (Freundlich, Langmuir, Temkin and Dubinin–Radushkevich) were used to match experimental data. The kinetic data fit well to a pseudo-second-order kinetic model. This implies that the availability of the adsorption sites is more important than the herbicide concentration. What is interesting is that the sorption of 2-hydroxyterbuthylazine was five times greater than desethyl-terbuthylazine. The Dubinin–Radushkevich isotherm model implied the physical character of the adsorption processes for both analytes, and the Temkin model proved their exothermic character. The investigation of adsorption capacities under dynamic conditions was also undertaken. This led to similar results to those seen in batch experiments. Furthermore, column studies showed that adsorbed metabolites of terbuthylazine can be simply desorbed by ethanol. It implies the possibility of multiple usages of investigated beads in adsorption processes, e.g., as a filling of SPE columns used as packing material for a chromatographic column dedicated to triazine herbicide measurements.

A study conducted by Kołodyńska et al. [4] proposes novel sorbents based on modified thermoplastic starch (TPS). The starting TPS materials were obtained from potato starch (containing 20% amylose) and 99.5% glycerine. In modification processes, poly(butylene succinate), polylactic acid, and hemp fibers were used. The newly obtained materials were employed for Cd(II) removal from Chinese herbal medicinal plant extracts, based on *D. officinale*. For detailed characterization of the TPS sorbents’ elemental analysis (CHN), infrared spectroscopy (ATRFTIR),thermogravimetry (TG), size-exclusion chromatography (SEC), a low-temperature nitrogen adsorption–desorption method, X-ray powder diffraction (XRD), scanning electron microscopy (SEM), and a point-of-zero-charge analysis (pH_pzc_ analysis) were used. Their adsorption properties were compared to properties of commercially available ion exchangers (Lewatit, Amberline and Puorite types). The effects of adsorption time, the initial concentration of Cd(II), and pH on the adsorption efficiency were investigated. The obtained results showed that materials based on TPS can be successfully applied in the purification of natural herbal extracts and can be used as a natural, cheap, efficient, and abundant adsorbent for further applications.
